# Epidemiological, clinical, therapeutic features and predictors of death among COVID-19 patients hospitalized in Parakou: a cross-sectional study in Northern Benin

**DOI:** 10.1186/s12879-023-08445-z

**Published:** 2023-07-20

**Authors:** Attinsounon Cossi Angelo, Yamongbè Clodel, Codjo Léopold, Adé Serge, Mama Cissé Ibrahim, Attinon Julien, Klikpezo Roger, Savi de Tovè Kofi-Mensa

**Affiliations:** 1 Infectious Diseases and Tropical Medicine Unit, Parakou, Benin; 2 Faculty of Medicine, University of Parakou, 03 P.O Box 112, Parakou, Benin; 3 Regional Teaching Hospital of Borgou, Parakou, Benin; 4 Regional Care Center of COVID-19 Cases of Parakou, Parakou, Benin; 5 Regional Military Hospital of Parakou, Parakou, Benin

**Keywords:** COVID-19, Epidemiology, Clinical features, Therapy, Prognosis, Parakou, Benin

## Abstract

**Background:**

COVID-19 is an emerging contagious infection with polymorphic clinical manifestations. The purpose of this study was to describe the epidemiological, clinical, therapeutic features and identify the predictors of mortality among COVID-19 hospitalized cases in Parakou.

**Methods:**

This was a cross-sectional, descriptive and analytic study. Systematic recruitment was used to include all patients hospitalized with COVID-19 from May 8, 2020, to December 31, 2021, whose medical records were available and usable. The variables studied were clinical and paraclinical signs, diagnostic and therapeutic means, evolution under treatment and prognostic factors. This study was approved by the Local Ethical Committee. The data were analyzed using Stata/MP 14.1 software.

**Results:**

A total of 198 cases of COVID-19 were identified, 117 of whom were men. The mean age was 51.53 ± 19.51 years. The presenting signs were fever 146 (74.11%), cough 157 (79.70%) and dyspnea 118 (53.90%). It was severe COVID-19 in 108 cases (54.55%). Therapeutically, 95 patients (47.98%) had received the combination of Lopinavir/ritonavir and Ribavirin and 95 others (47.98%) received chloroquine. Recovery was noted in 151 (76.26%) patients. Mortality rate was 18.18%. Predictors of death were high blood pressure, presence of signs of severity, high-concentration mask ventilation used, and elevated transaminases.

**Conclusion:**

COVID-19 was a reality in Parakou, with a significant number of severe cases requiring hospitalization. Several factors are associated with the prognosis of the disease.

## Background

The second coronavirus infection causing severe acute respiratory syndrome named the coronavirus infectious disease of 2019 (COVID-19) started in Wuhan, Hubei province of China as an outbreak in December 2019 [1, 2]. This is the third coronavirus epidemic after SARS-CoV in 2002–2003 and Middle East Respiratory Syndrome (MERS) in 2012 [3]. This disease, due to its rapid spread, very early led to a global health crisis [4]. Thus, it was declared on March 11, 2020 as a pandemic by the World Health Organization (WHO) [5].

This new disease, whose clinical manifestations are polymorphic and non-specific, ranging from a simple flu-like syndrome to a severe and fatal pneumonia, has made even the most developed countries experience a real health nightmare [6, 7].

COVID-19 has also been the disease of all scientific controversies, whether diagnostic, preventive or curative. Witness the controversy surrounding the efficacy of masks [8, 9], the efficacy of different diagnostic methods [10, 11] and the great controversy surrounding the efficacy of hydroxychloroquine, Ivermectin and other therapeutic methods [12, 13]. Benin, after recording its first case of COVID-19 in March 2020, faced successively the first, second, third and fourth wave of the epidemic with 27,638 confirmed cases including 163 deaths in December 2022 [14]. Since the first wave, several measures have been taken to control the epidemic, including the creation of sites for the management of severe cases. Thus, the Army Training Hospital (HIA) in Parakou was transformed into an isolation and management center for COVID-19 cases.

Despite the controversy surrounding the treatment of the disease, Benin's Ministry of Health set up a committee of experts to propose a therapeutic protocol. Patients with severe COVID-19 were treated with antivirals (lopinavir/ritonavir and ribavirin) available only in treatment centers. For patients with simple or moderate forms of the disease, chloroquine and azithromycin were the first-line treatments. Also, treatment centers have been equipped with a significant capacity for medical and respiratory resuscitation in order to improve the prognosis of admitted patients.

After two years of hospital management of this pandemic, and in the context described above, characterized by the implementation of an empirical protocol, we felt it necessary to take stock of this management in one of the country's main sites, to assess the results obtained and to draw lessons for future times. This is the rationale behind the present study, whose objectives were to describe the epidemiological, clinical, therapeutic features and identify the predictors of mortality among COVID-19 hospitalized cases in Parakou.

## Patients and methods

### Study setting and patient care modality on the site

The study took place at the Parakou Epidemic Isolation and Treatment Center, located in the Borgou Department in northern Benin. This center was built within the Armed Forces Training Hospital, whose routine activities were suspended for the occasion. The premises have been refurbished and adapted to the management of COVID-19 cases.

Patients are classified into three categories. Simple cases were paucisymptomatic patients with no comorbidities and no high-risk terrain. Moderate cases were symptomatic patients with comorbidities or at-risk terrain, but who were stable and had no organ dysfunction. Finally, severe cases were patients with respiratory instability (dyspnea, hypoxemia, respiratory distress), hemodynamic instability and/or severe organ dysfunction. This center was the only one in northern Benin and received patients from 5 departments of the country, namely Borgou, Alibori, Atacora, Donga and Collines. Thus, all patients who did not meet the criteria for self-containment were hospitalized there. These were either severe cases (respiratory instability, hemodynamic instability, severe organ dysfunction, etc.) or cases that could not be treated orally, or any situation requiring hospitalization.

The team of caregivers formed for the occasion was composed of an infectious disease specialist, an intensive care specialist and anaesthetist, a cardiologist, a pneumologist, a psychologist, four general practitioners, paramedics (nurses, nurses' aides, hygienists). Other specialists are called upon as needed for occasional interventions.

The center had at its disposal the biomedical analysis and medical imaging services of the hospital that housed it.

This laboratory was able to carry out diagnostic tests (blood count, CRP), basic microbiological tests (cytobacteriological tests, blood cultures), various serological tests (HIV, viral hepatitis, etc.) and other biological tests (ionogram, renal, hepatic, etc.).

The imaging department offered the possibility of performing thoracic angioscans on site. This examination graded lung parenchymal involvement into 5 stages based on the percentage of lung damaged: absent or minimal involvement (< 10%), moderate (10–25%), extensive (25–50%), severe (50–75%) or critical (> 75%). The French Society of Radiology has also classified lesions into three categories: lesions suggestive of COVID-19 (ground-glass opacities or consolidation with bilateral character, peripheral extension and distribution, absence of pleural effusion), lesions compatible with COVID-19 (single alveolar condensation, image of bronchiolitis, etc.) and lesions not suggestive of COVID-19 (non-infectious lesions) [15].

Patients are referred to the site on the basis of a positive test for COVID-19. In the majority of cases, this was PCR or antigenic testing. On admission, a confirmatory PCR test is performed on all patients as soon as they are admitted to hospital. Patients with a negative PCR for COVID-19 were referred to the departmental university hospital in Borgou for standard care.

Therapeutically, the majority of patients referred to the site had already started chloroquine 250 mgx3/d and azithromycin 500 mg D1, 250 mg/d from D2—D5 prior to admission. The management protocol for severe cases was based on Lopinavir/Ritonavir (800/200 mg × 2/d) orally, Ribavirin 400 mg × 2/d IV, Dexamethasone injection 8—12 mg/d (in case of dyspnea) and Enoxaparin in preventive or curative dose depending on the risk of thromboembolic events. The protocol provided for the systematic initiation of antibiotic therapy in all severe cases. This consists of either azithromycin 500 mg on Day 1, 250 mg/d from Day 2 to Day 5, or the combination of amoxicillin and clavulanic acid 1gx3/d, or ceftriaxone 2 g/d. The clinical staff can modify this protocol according to the particularities of each patient. An adjuvant treatment is often systematically instituted. These include vitamin C, zinc, calcium, hydro electrolytic support, and omeprazole. Other specific or symptomatic treatments are usually added according to the patient's needs.

### Type of study

This was a cross-sectional, descriptive, analytical study with retrospective data collection.

### Study population

The study population consisted of all patients hospitalized for COVID-19 in Parakou.

### Inclusion criteria

All patients hospitalized and managed for PCR based confirmed COVID-19 between May 7, 2020 and December 31, 2021 were included in the study.

### Exclusion criteria

Patients who died on admission and patients whose medical records were unavailable or not usable because of lack of information were excluded from the study.

### Sampling

This was a comprehensive census of all patients admitted to the site during the study period who met the inclusion criteria.

### Study variables

#### Dependent variable

To investigate prognostic factors of the disease, the occurrence of death was considered as the dependent variable in the data analysis.

#### Independent variables

The independent variables were epidemiological data (evolution of cases over time, epidemic peak periods), sociodemographic (age, sex, origin, marital status), clinical (comorbidities, pathological history, clinical signs, signs of severity, time to consultation), paraclinical (organ dysfunctions, biological abnormalities, radiological abnormalities), therapeutic (antiviral or antiretroviral drugs, chloroquine, azithromycin, duration of treatment, stay in intensive care unit) and evolutionary (complications, recoveries, deaths) data.

### Data collection

#### Collection tool

An input mask was developed on the Kobotoolbox platform. The collection grid was therefore electronic. A pre-test was carried out and allowed to correct the insufficiencies.

#### Collection process

A manual search of all medical records of patients hospitalized for covid-19 during the study period was performed. The necessary information was therefore extracted from each patient's chart and then integrated into the KoboCollectv2022.1.2 software.

### Data analysis

Only data relating to the subjects included were entered into the database.

After checking the completeness of the data entered in the KoboCollectv2022.1.2 software, the data analysis was done with the Stata/MP 14.1 software. The text processing and the preparation of the tables were carried out with Microsoft Word and Excel version 2019 application software. The qualitative variables were expressed in number and percentage and the quantitative variables in mean ± standard deviation. The analytical phase consisted of the search for association between the independent variables and the occurrence of death. The measure of association used was the odds ratio (OR). Chi-square or FISCHER statistical tests were used as appropriate to determine the degree of significance of the association (*p*-value). The significance level was set at 0.05. After univariate analysis, variables significantly associated with death were included in a multivariate model. Their degree of association was estimated using the adjusted odds ratio.

### Ethical considerations and good practices

This study was approved by the Local Ethical Committee for biomedical research of the University of Parakou (REF 0559/CLERB-UP/P/SP/R/SA of 31^th^ January 2022).

The data were treated confidentially and anonymously. All the methods included in this study are in accordance with the declaration of Helsinki. Informed consent has obtained from all patients upon admission to the center.

## Results

Of the 205 patients admitted to the site during the study period, biological evidence of SARS-COV-2 infection was found in 198 patients with usable records. Seven patients were therefore excluded, and the study covered 198 patients.

### Epidemiological curve of covid-19 cases recorded at the site during the study period

**Fig. 1:**
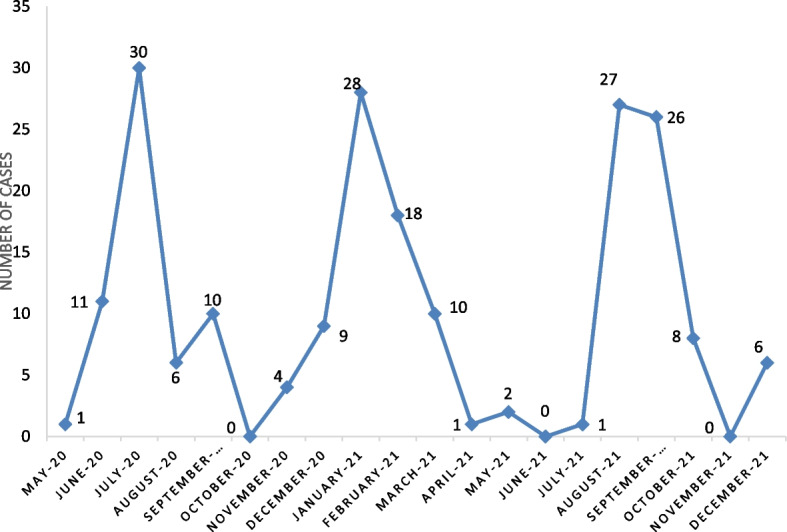
Distribution of COVID-19 cases by month of onset, Parakou from 2020–2021

From May 2020 to December 2021, three epidemic peaks were noted according to the evolution of the number of cases admitted to the site, in July 2020, January 2021 and July 2021 respectively (Fig. 1).

### Socio-demographic characteristics of patients

Of the 198 patients included, 117 (59.09%) were male with a sex ratio of 1.44. The mean age was 51.53 ± 19.51 years. Patients aged at least 50 years were the most represented (52.53%) (Table 1).

**Table 1:** Socio-demographic characteristics and treatment received among COVID-19 patients hospitalized in Parakou from 2020–2021 (*N* = 198)

Socio-demographic characteristics				Treatment		
	N	%			N	%
Age (years old)	198	100	Antiviral			
≤ 25	26	13.13	Lopinavir/ritonavir/ribavirin		95	47.98
[25–50]	68	34.34	Lopinavir/ritonavir		28	14.14
[50–75]	83	41.92	Chloroquine + azithromycin		24	12.12
> 75	21	10.61	Oxygenation			
Gender	198	100	Intubation		03	1.52
Male	117	59.09	Non-invasive ventilation		60	30.30
Female	81	40.91	H-C mask oxygenation		87	43.94
Occupation	198	100	Concentrator Oxygenation		61	30.81
Employees	60	30.30	Antibiotic			
Freelance workers	59	29.80	Azithromycin		181	93.78
Health workers	10	5.05	C3G and/or metronidazole		107	54.04
Students	8	4.05	Other treatments			
Unspecified	61	30.80	Dexamethasone		137	69.19
Marital status	198	100	Enoxaparin		146	73.41
In couple	145	73.23	Anxiolytics		42	21.21
Single	32	16.16				
Not specified	21	10.61				

*C3G* Ceftriaxone or Cefotaxime, *H-C* High-Concentrator

### Clinical and diagnostic features

The mean time to admission of patients to hospital after the onset of symptoms was 8.84 ± 8.48 days. Sixty-one (30.81%) patients were admitted more than 10 days after the onset of symptoms.

The three main signs found in patients were cough (157 cases; 79.70%), asthenia (157 cases; 79.70%) and fever (146 cases; 74.11%). Dyspnea was present in 118 patients (53.90%). Table 2 shows the symptoms recorded on admission of patients to the management site.

**Table 2:** Symptoms presented on admission and comorbidities among COVID-19 patients hospitalized in Parakou from 2020–2021 (*N* = 198)

Symptoms			Comorbidities		
	N	%		N	%
Cough	157	79.70	High Blood Pressure	66	33.50
Asthenia	157	79.70	Diabetes	45	22.84
Fever	146	74.11	Obesity	20	10.15
Dyspnea	118	53.90	Asthma	13	6.60
Headaches	79	40.10	Heart failure	18	9.09
Expectoration	24	12.12	Viral hepatitis B and C	17	8.63
Myalgia	57	28.93	Smoking	10	5.08
Anorexia	57	28.93	HIV	9	4.57
Rhinorrhea	54	27.41	Sickle cell disease	7	3.55
Anosmia	48	24.37	Pregnancy	7	3.55
Agueusia	47	23.86	Cancer	7	3.53
Nausea/vomiting	32	16.16	Renal failure	6	3.05
Chest pain	32	16.16	Chronic alcoholism	5	2.54
Diarrhea	26	13.20	Tuberculosis	3	1.52
Sore throat	20	10.10	COPD	3	1.52
Abdominal pain	11	5.56	No comorbidity	45	22.84
Coma	8	4.04	Other comorbidities^b^	3	1.51
Hemoptysis	7	3.54			
Palpitation	5	2.53			
Arthralgia	5	2.53			
Other^a^	7	3.54			

*HIV* Human Immunodeficiency Virus, *COPD* Chronic obstructive pulmonary disease

^a^Dysphonia, Insomnia, Hiccups, Pruritus, Paresthesia, weight loss, visual disturbances

^b^G6PD deficiency, Obstructive sleep apnea syndrome, Wolf Parkison syndrome

A comorbidity was present in 152 patients (76.77%). There were 66 cases (33.50%) of hypertension and 45 cases of diabetes (22.84%). Other comorbidities are presented in Table 2.

Diagnostically, confirmation of COVID-19 was based on molecular biology (PCR) in 159 patients (88.83%), based on antigenic testing in 83 patients (88.30%).

Of the 63 patients who had chest CT scans, lung abnormalities had been noted in 60 patients (95.24%). The lesions were typical of COVID-19 in 32 patients (50.79%), compatible with COVID-19 in 18 patients (28.57%) and non-evocative or normal in 13 patients (20.64%). It was critical, severe or extensive in 37 patients (58.73%) and minimal or moderate in 19 patients (30.16%).

### Immunization status of patients

Six patients (3.03%) were vaccinated, three with Johnson & Johnson vaccine and three with Sinovac vaccine.

### Signs of severity and organ dysfunction on admission

On admission to the treatment site, 103 (52.02) patients had at least two organ dysfunctions. Respiratory distress was noted in 164 patients (82.83%). There were 49 cases of hepatic dysfunction (24.75%) including 38 cases (77.77%) of hepatic cytolysis, 30 cases (15.15%) of renal dysfunction, 29 cases (14.65%) of cardiocirculatory dysfunction and eight cases (4.04%) of neurological dysfunction.

Biologically, CRP was greater than or equal to 96 mg/L in 145 patients (73.23%). Hyperglycemia was recorded in 102 patients (52.02%). Severe anemia was present in 68 patients (34.34%) and 56 patients (28.28%) had hypercreatinemia with 42 cases (21.21%) of hyperuricemia. Cases of thrombocytopenia (28 or 14.14%), hyperleukocytosis (64 or 32.32%) and lymphopenia (96 or 48.48%).

### Therapeutic characteristics

According to the case management protocol, 95 patients (47.98%) had received the combination of Lopinavir/ritonavir and Ribavirin. Of the 95 (47.98%) patients admitted to the site on chloroquine + azithromycin, this treatment was continued in 24 (12.12%). A total of 181 patients (93.78%) received at least one antibiotic and 137 (69.19%) were put on dexamethasone. Table 1 shows the treatment received by the patients at the site.

### Evolutionary characteristics

During hospitalization, 160 patients (80.81%) had at least one complication. These were renal failure (83 cases; 41.82%), severe hyponatremia (17 cases; 8.56%), severe hypokalemia (19 cases; 9.60%), hypocalcemia (18 cases; 9.09%), severe anemia (28 cases; 14.14%), hepatic cytolysis (24 cases; 12.12%), cardiac decompensation (19 cases; 9.60%), digestive disorders (18 cases; 9.09%), respiratory distress (5 cases; 2.53%) and coma (3 cases; 1.52%).

The average length of hospitalization for patients was 11.96 ± 4.22 days.

Regarding the outcome of the patients, 151 patients (76.26%) were completely cured, 10 patients (5.05%) were released from isolation after a negative PCR, one patient was transferred to the Allada site in the south of Benin and 36 patients died, i.e., a hospital mortality of 18.18%.

### Poor prognostic factors

Factors associated with death in univariate analysis are reported in Table 3.

**Table 3:** Predictors of death in univariate analysis among COVID-19 patients hospitalized in Parakou from 2020–2021 (*N* = 198)

	Deaths				RC^a^ [IC^b^ (95%)]	*P*^c^
	Yes		No			
	N	%	N	%		
General condition						< 0.01
Good	1	2.27	43	97.73	1	
Altered	35	22.73	19	77.27	12.65[1.68–95.16]	
Age						0.006
< 50 years	9	10.11	80	89.89	1	
≥ 50 years	27	24.77	82	75.23	2.93[1.30–6.61]	
Heart failure						0.01
Yes	6	46.15	7	53.85	4.40[1.38–14.01]	
No	30	16.30	154	83.70	1	
Oxygen saturation						< 0.01
SpO_2_ < 95	32	28.83	79	71.17	11.20[3.29–38.06]	
SpO_2_ $\ge$ 95%	3	3.49	83	96.51	1	
High blood pressure						< 0.01
Yes	14	42.42	19	57.58	4.72[2.07–10.76]	
No	22	13.50	141	86.50	1	
Clinical severity						< 0.01
Simple/moderate	1	1.32	75	98.68	1	
Grave	33	30.56	75	69.44	33[4.40–247.53]	
Aspartate aminotransferase						< 0.01
Normal	7	6.73	97	93.27	1	
High	25	31.65	54	68.35	6.42[2.60–15.81]	
Alanine aminotransferases						< 0.01
Normal	19	14.07	116	85.93	1	
High	13	27.08	35	72.92	2.28[1.01–5.05]	
Total hypocholesterolemia						< 0.01
Yes	25	28.09	64	71.91	5.23[1.88–14.50]	
No	5	6.94	67	93.06	1	
HDL hypocholesterolemia						0.003
Yes	23	27.06	62	72.94	3.60[1.45–8.98]	
No	7	9.33	68	90.67	1	
Creatinine levels						0.003
Normal	15	12.82	102	87.18	1	
High	15	28.30	38	71.70	3.23[1.47–7.07]	
Number of failed organs						< 0.01
0 or 1	4	4.30	89	95.70	1	
˃1	32	30.48	73	69.52	9.75[3.30–28.85]	
Antivirals						< 0.01
Yes	29	28.16	74	71.84	4.93[2.04–11.89]	
No	7	7.37	88	92.63	1	
Dexamethasone						< 0.01
Yes	33	24.09	104	75.91	6.13[1.80–20.87]	
No	3	4.92	58	95.08	1	
Enoxaparin						0.01
Yes	32	21.91	114	78.08	3.37[1.13–10.05]	
No	4	7.49	48	91.81	1	
Oxygenation by mask^a^						< 0.01
Yes	33	37.93	54	62.07	22[6.45–77.98]	
No	3	2.70	108	97.30	1	
Non-invasive ventilation						< 0.01
Yes	22	36.37	38	63.33	5.13[2.39–10.99]	
No	14	10.14	124	89.86	1	
Oxygen concentrator						0.40
Yes	9	14.75	55	85.25	0.70[0.31–1.66]	
No	27	19.71	110	80.29	1	
Gender						0.78
Female	14	17.28	67	82.72	1	
Male	22	18.80	95	81.20	1.11[0.53–2.32]	
Comorbidity						0.14
Yes	31	20.26	122	79.24	2.03[0.74–5.58]	
No	5	11.11	40	88.88	1	
Diabetes						0.23
Yes	11	24.44	34	75.56	1.64[0.74–3.67]	
No	25	16.45	127	83.55	1	
Overweight/obesity						0.42
Yes	5	25.00	15	75.00	1.57[0.53–4.64]	
No	31	17.51	146	82.49	1	

^a^*RC* odds ratio

^b^*IC (95%)* 95% confidence interval

^c^*p*-value of wald’s chi-square

In multivariate analysis, high blood pressure, elevated aspartate aminotransferase transaminases, severity of clinical presentation, and use of high-concentration mask oxygenation were associated with death (Table 4).

**Table 4:** Predictors of death in multivariate analysis among COVID-19 patients hospitalized in Parakou from 2020–2021

	Deaths		RC _brut_ [IC]^‡^_(95%)_	_Adjusted_ OR^¶^ [CI]^‡^_(95%)_	*p*-value^†^
	N	%			
High blood pressure					0.006
Yes	16	24.24	1.78[0.85–3.71]	4.16[1.47–11.72]	
No	20	15.27	1		
Aspartate aminotransferase					0.02
Normal	7	6.73	1	3.61[1.24–10.53]	
High	25	31.65	6.42[2.60–15.81]		
Mask oxygen therapy					0.002
Yes	9	14.75	22[6.45–77.98]	0.23[0.08–0.62]	
No	3	2.70	1		
Gravity					< 0.01
Not serious	1	1.32	1	36.90[4.39–310.44]	
Grave	33	3.56	33[4.40–247.53]		

^*^RC odds ratio

^†^*IC (95%)* 95% confidence interval

⁋*p*-value of wald’s chi-square

## Discussion

The aim of this study was to describe the epidemiological, clinical, therapeutic and evolutionary aspects of patients hospitalized with COVID-19 in North Benin.

### Epidemiological aspects

Epidemiologically, the average age of the patients included in the present study was 51.53 years. Although COVID-19 affects all individuals regardless of age, it is also known that there is a strong relationship between age and disease severity [16, 17]. Thus, in several studies, hospitalized patients were generally older than 50 years [18]. In contrast, in studies including all cases in general, including outpatients, paucisymptomatic or asymptomatic cases, the median and mean ages are not as high and are around 30 years old [19, 20] which reflects the wide distribution of SARS-CoV2 in young subjects.

It is also true that age-related vulnerability is influenced by the level of development in general and the health system in particular in each country.

With regard to gender, a male predominance of 59.09% was noted in Parakou. Indeed, several studies have noted a male predominance either among symptomatic cases or among severe cases and therefore hospitalized [19, 21]. This male susceptibility to SARS-CoV-2 infection could be explained by a difference in innate immunity between men and women. Female gender has been shown to have a better cellular and humoral immune response against viral infections compared to male gender. The female X chromosome and hormonal factors are thought to contribute to the reduced vulnerability of women to viral infections [22].

### Clinical and paraclinical manifestations

SARS-COV-2 is known for its strong tropism for the respiratory system. However, the clinical manifestations are multiple and their frequency varies according to the studies. Some symptoms seem to be mandatory. These are fever, cough, asthenia, dyspnea and headache [16, 23]. Digestive disorders, anosmia, agueusia, myalgias and rhinorrhea are found with varying frequencies from one study to another [24]. It is not excluded that the patient insists only on the symptoms that concern him.

Biologically, in the present study, several organ dysfunctions and biological abnormalities were noted in the inflammatory (very high CRP, neutrophilia, severe lymphopenia) and metabolic (hyperglycemia, hepatic cytolysis, hypercholesterolemia) fields. Severe anemia requiring blood transfusion was regularly recorded, and was a factor aggravating hypoxemia. Renal insufficiency was also present, with elevated creatinine levels in a significant proportion of patients. All these biological abnormalities reflect the importance of the metabolic and organic disorders caused by SARS-CoV2 infection. All these organ dysfunctions and biological abnormalities have been documented in the literature. This is the case for the considerable elevation of D-dimer, a marker of a thromboembolic disorder [25, 26].

### Case management

Benin has developed a treatment protocol that is based on chloroquine combined or not with azithromycin for patients treated on an outpatient or self-isolation basis. This protocol is therefore reserved for simple or moderate cases not requiring hospitalization. The hospitalized patients benefited from a protocol based on antiretrovirals (lopinavir/r) and an antiviral (ribavirin). The latter protocol is reserved for severe cases, particularly patients with dyspnea or hypoxia. The rest of the management is based on infectious and medical resuscitation according to the clinical presentation of each patient. Thus, 52.02% of patients received Ribavirin associated with Lopinavir/ritonavir and 47.98% were treated with Chloroquine. But it should be noted that most of the patients start azithromycin and chloroquine before their admission to the management center. Some continue this treatment during their hospitalization, if not, it is replaced by the so-called severe case protocol. Indeed, several West African countries have adopted chloroquine and azithromycin as first-line treatment for COVID-19 [23, 27–29]. The efficacy of this protocol is highly controversial. While several studies have shown its benefit in terms of mortality reduction [30, 31], others question its benefit [32, 33].

In this study, corticosteroid therapy (dexamethasone) was instituted in all patients with dyspnea. Zinc and vitamin C are also used to reinforce the patients' immunity. Several therapeutic trials were conducted and treatment protocols were developed in several countries as the results of these trials appeared to be conclusive. In Kenya, for example, Remdesivir, tocilizumab and dexamethasone were chosen [34]. This is also the case for the use of statins as lipid metabolism moderators, anti-inflammatory, antithrombotic agents and immunomodulators [35].

Indeed, never before has the treatment of a disease been so controversial in the world.

### Evolution and prognostic factors

The specificity of the SARS-COV-2 virus is its ability to induce acute respiratory distress that can rapidly become life threatening. In addition, there are numerous organ dysfunctions. Each patient passes this ordeal according to his intrinsic risk factors and the quality of the care he receives. This justifies the great variability in hospitalization times and mortality noted in the literature.

In the present study, the average length of hospital stay was 11.96 days. It was 14 days in Ethiopia [36]. In China, on the other hand, the length of hospitalization of patients ranged from 12.8 days to 21 days [37, 38]. This difference could be explained by the severity of the clinical condition of the patients on admission, the existence of comorbidity with a susceptibility to decompensation and the occurrence of complications during hospitalization responsible for admissions to intensive care units.

In the present study, mortality was 18.18%. Mortality was 2.6% in Lagos (Nigeria) [24] and 9.28% in Sikasso (Mali) [28]. On the other hand, very high mortalities have been recorded with 52.4% in China [7] and 48.3% in Italy [39].

Indeed, Africa has had one of the lowest mortality rates. The difference is clear when comparing the mortality of African countries to that of other countries, especially those of developed countries. This observation raises several hypotheses relating to the youth of the African population, climatic, genetic and environmental factors. What is certain is that the African population presents to a lesser extent the severity and mortality factors of COVID-19.

In the present study, factors associated with death were high blood pressure, elevated aspartate aminotransferases, high concentration mask oxygenation, and severe COVID-19. Several factors influence the prognosis of this disease. These factors are individual, but also related to the performance of the health system, the technical platform, the organization of care, and the capacity of the hospital. Many prognostic factors have been identified in the literature [17, 25, 26, 36, 39]. This variability of factors associated with death from one study to another could be explained by the diversity of the populations studied, but also by the difference between the variables studied.

### Strengths and limitations of the study

This study has the merit of giving a preliminary idea of the epidemiological, clinical, therapeutic and prognostic characteristics of patients with COVID-19 hospitalized in northern Benin. It could therefore contribute to a better understanding of the profile of severe cases and improve the response and the quality of management of these cases.

However, like any retrospective study, its main weakness lies in the lack of information on certain epidemiological and clinical data. The context of medical emergencies also did not always allow the medical observations to be filled in properly.

## Conclusion

In conclusion, COVID-19 was a reality in Parakou, with a significant number of severe cases requiring hospitalization. Chloroquine, Lopinavir/ritonavir with or without ribavirin, and azithromycin-based antibiotics were the main treatments received by patients, leading to recovery in more than four out of five cases. Several factors are associated with the prognosis of the disease, particularly high blood pressure and the severity of the disease requiring artificial ventilation. Taking these factors into account will help to better organize the response in Benin and improve the prognosis of the disease.

## Data Availability

All data generated or analysed during this study are included in this published article.

## abbreviations

COVID-19: Coronavirus Diseases 2019
CT Scan: Computed Tomography Scan
PCR: Polymerase Chain Reaction
WHO: World Health Organization
CRP: C-reactive protein
IV: Intravenous
OR: Odds ratio
D: Day
SARS-CoV2: Severe Acute Respiratory Syndrome Coronavirus 2
